# Metabolites and lipid species mediate the associations of adiposity in childhood and early adulthood with mammographic breast density in premenopausal women

**DOI:** 10.1186/s13058-025-01970-6

**Published:** 2025-02-04

**Authors:** Kayla R. Getz, Myung Sik Jeon, Lili Liu, Lei Liu, Haixiang Zhang, Chongliang Luo, Jingqin Luo, Adetunji T. Toriola

**Affiliations:** 1 Division of Public Health Sciences, Department of Surgery, Washington University School of Medicine, 660 South Euclid Avenue, Box 8100, St. Louis, MO USA; 2 Siteman Cancer Center Biostatistics Shared Resource, Division of Public Health Sciences, Department of Surgery, Washington University School of Medicine, St. Louis, MO USA; 3 Division of Biostatistics, Washington University School of Medicine, St. Louis, MO USA; 4 Center for Applied Mathematics, Tianjin University, Tianjin, 300072 China; 5 Siteman Cancer Center, Washington University School of Medicine, St. Louis, MO USA

**Keywords:** Early-life, Age 10, Age 18, Body mass index, Lipidomics, Mammographic breast density, Metabolomics, Premenopausal

## Abstract

**Background:**

Mammographic breast density (MBD), a strong predictor of breast cancer, is highly influenced by body mass index (BMI) in childhood and early adulthood, but the mechanisms underlying these associations are not fully understood. Our goal is to identify biomarkers that mediate the associations of BMI at ages 10 and 18 with MBD in premenopausal women.

**Methods:**

This study consists of 705 premenopausal women who had their screening mammogram at Washington University in St. Louis, MO, and provided a fasting blood sample. Our comprehensive metabolomic and lipidomic profiling yielded complete data for 828 metabolites and 857 lipid species after imputation. We used Volpara to determine volumetric measures of MBD. We performed high dimensional mediation analysis using the *HIMA* R package, adjusted for confounders, to determine whether lipid species and metabolites mediate the associations of BMI at 10 and 18 with MBD. We applied a false discovery rate (FDR) *p*-value < 0.1.

**Results:**

Four metabolites (glutamate, β-cryptoxanthin, cortolone glucuronide (1), phytanate) significantly mediated the association of BMI at 10 with volumetric percent density (VPD), and two (glutamate, β-cryptoxanthin) mediated the association of BMI at 18 with VPD. Glutamate was the strongest mediator across time points. Glutamate mediated 6.7% (FDR *p*-value = 0.06) and 9.3% (FDR *p*-value = 0.008) of the association between BMI at age 10 and 18, respectively. Four lipid species (CER(18:0), LCER(14:0), LPC(18:1), PC(18:1/18:1)), mediated the association of BMI at 10 with VPD, while five lipid species (CER(18:0), LCER(14:0), PC(18:1/18:1), TAG56:5-FA22:5, TAG52:2-FA16:0) mediated the association of BMI at 18 with VPD. The strongest mediator was PC(18:1/18:1), which mediated 9.7%, (FDR-*p* = 0.009) and 7.7%, (FDR-*p* = 0.04) of the association of BMI at age 10 and 18 with VPD, respectively.

**Conclusions:**

Metabolites in amino acid, lipid, cofactor/vitamin, and xenobiotic super-pathways as well as lipid species across the phospholipid, neutral complex lipid and sphingolipid super-pathways mediated the associations of BMI in early-life and MBD in premenopausal women. This study offers insight into the biological mechanisms underlying the link between early-life adiposity and MBD, which can support future research into breast cancer prevention.

## Background

Early-life and adulthood body mass index (BMI) are inversely associated with both mammographic breast density (MBD) and breast cancer among premenopausal women [1–3]. BMI explains the most variation in MBD (26%) compared to other well-established risk factors [4]. Nevertheless, the underlying biological mechanisms explaining the association of early-life BMI and MBD have not been well characterized. Understanding the underlying biological mechanism can support research into breast cancer prevention early in life.

Metabolomics provides an expansive representation of many exposures and may imply phenotype [5]. The metabolome is also associated with various measures of adiposity. Studies have identified positive associations between glutamate and mannose with BMI, as well as inverse associations between β-cryptoxanthin [6–8]. Similarly, the lipidome, which provides an overall representation of lipid metabolism and a detailed overview of heterogeneous functions of lipids, is also strongly related to adiposity [9]. The lipidome is associated with BMI. Phospholipids, particularly species from the lysophosphatidylcholine (LPC) sub-pathway, are inversely associated with BMI, whereas neutral complex lipid species from the largely enriched triacylglycerol (TAG) sub-pathway are often positively associated with BMI [10–14].

Studies have reported associations of the lipidome and various metabolites, especially in amino acid, vitamin, and related cofactor sub-pathways with MBD in premenopausal women [15–17]. Given the associations of the metabolome/lipidome with BMI as well as those between metabolites/lipid species with MBD; it is possible that the associations of childhood/early adulthood BMI with MBD is mediated by metabolites and lipid species. Yet, only one small study in 182 women aged 25–29 years has explored the possible role of metabolites in mediating the association of childhood adiposity with MBD. The study also utilized a traditional mediation analysis approach [18].

Our goal in this study is to perform high-dimensional mediation analyses to identify lipid species and metabolites that mediate the associations of childhood/early adulthood BMI with MBD in premenopausal women who were recruited during screening mammogram.

## Methods

### Study population

This study is comprised of 705 premenopausal women who attended the Joanne Knight Breast Health Center at Washington University School of Medicine in St. Louis, MO, for their screening mammogram and provided a blood sample for lipidomics and metabolomics profiling. Women were not eligible to participate in the study if they were postmenopausal, which was determined as not having had a menstrual period in the past 12 months, having a history of taking hormone replacement therapy, or had their ovaries surgically removed [2, 3]. We excluded premenopausal women who had a personal history of cancer, breast augmentation (reduction or implants), who were pregnant or had used selective estrogen receptor modulators in the past six months [2, 3]. On the same visit as the screening mammogram, women provided a fasting blood sample that was immediately sent to the Tissue Procurement Core at Siteman Cancer Center, where it was stored at − 80 °C [19]. All participants provided written informed consent, and the study was conducted within the guidelines of the Declaration of Helsinki. The study was approved by the institutional review board at the Washington University School of Medicine, St. Louis, MO.

### Anthropometric and self-reported measures

Trained research staff measured anthropometric characteristics at enrollment. Height was measured using a stadiometer, and we measured weight using the OMRON Full Body Sensor Body Composition Monitor and Scale Model HBF-514FC [2, 3]. Additionally, participants were asked to complete a questionnaire that provided information on their demographics, reproductive/family history and adiposity measures from childhood and early adulthood. Adiposity at age 10 was assessed utilizing the Stunkard pictogram and converted to BMI based on values generated from the Growing Up Today Study [20]. BMI at age 18 was calculated from self-reported weight at age 18 and height at enrollment. Women who reported a history of breast cancer diagnosis in either a mother or a sister were classified as having a positive family history of breast cancer.

### Lipidomic/metabolomic profiling

Metabolon (Durham, NC) performed untargeted lipidomics profiling and measured 982 individual lipid species. The process of how the lipid species were quantified and the reproducibility of the measures has been previously published [17, 21]. Of the 982 lipid species, 125 species were excluded due to excess missing observations (in > 300 samples). Lipid species with missing observations in < 300 samples were imputed using the 10-nearest neighbor method [22]. We had complete data available after imputation for 857 lipid species. Metabolon (Durham, NC) also performed untargeted metabolomic profiling on samples from the same women utilizing ultrahigh performance liquid chromatography/mass spectrometry (UHPLC/MS) [23]. A recently published article from our lab provides a detailed description of the methodology and process of UHPLC/MS as well as the quality control techniques performed in this study population [15]. Through the untargeted metabolomic profiling the global assay was able to detect 1,074 metabolites, but we excluded 246 metabolites because they were missing > 300 observations. Metabolites missing < 300 observations were imputed using the 10-nearest neighbor methods [22]. Imputations for metabolites and lipids were done separately using the “impute” package in R and using the 10-nearest neighbor method (kNN) which uses a Euclidean metric to identify the 10 nearest neighbors, then averages the values from those observations to impute the missing value [24]. There were very few missing observations (in total 0.4% for lipids and 1.3% for metabolites) since we excluded metabolites or lipid species with > 300 missing observations. To mitigate batch effect, peak area metabolite data were normalized using ComBat [25]. Since we were provided with concentrations for the lipid species data we did not normalize with ComBat. Lipid species concentrations and metabolites peak area data were log_10_ transformed to improve homoscedasticity and were standardized to reflect a unit change equal to the standard deviation of the lipid species/metabolite.

### Mammographic breast density measurement

Mammographic breast density was measured volumetrically using Volpara version 1.5. Volumetric percent density (VPD) was quantified by dividing maximum dense volume cm^3^ (DV), represented as the fibroglanduar tissue in the breast by total breast volume. Non-dense volume cm^3^ (NDV) is calculated by subtracting the amount of DV from the total breast volume. Volpara identifies the amount of fibroglanduar tissue in the breast, by taking the maximum value between the mediolateral oblique and cranial-caudal views of both breasts. Of the 705 women in the study, we were able to calculate MBD for 700, which was the final analytic sample. All measures of MBD were log_10_ transformed for analysis to conform to the normality assumption.

### Statistical analyses

We examined the distribution of BMI at ages 10 and 18 by presenting the means and standard deviations across age at enrollment, age at menarche, race, and family history of breast cancer. We performed multivariable linear regression to assess the associations between BMI at ages 10 and 18 across all lipid species and metabolites. We adjusted for age (continuous), race (non-Hispanic white, non-Hispanic black, other), and family history of breast cancer (yes, no) when investigating the associations between BMI at age 10 and the lipid species/metabolites. We additionally adjusted for age at menarche (continuous) and BMI at age 10 when assessing the relationship between BMI at age 18 and the lipid species/metabolites. Although there was very little missingness across variables (< 12%), if the BMI measures or any covariate were missing observations, they were imputed using multivariate imputation by chained equations [26]. There were also very few missing values for BMI at age 10 (N = 43) BMI at age 18 (N = 3).

Based on this a priori knowledge, we determined whether lipid species and metabolites mediate the associations of BMI at age 10 and BMI at age 18 with MBD through a high dimensional mediation analysis (HIMA) using the *HIMA* R package [27]. HIMA performs mediator screening by calculating the marginal correlations between mediators (metabolites or lipid species) and outcome (MBD) after adjusting for confounders. HIMA requires that all the mediators are included in the model at the same time (metabolite models include metabolites N = 828 and lipid species models include lipid species N = 857). It relies on sure independent screening and minimax concave penalty techniques to simultaneously incorporate multiple mediators, and uses a joint significance test for their mediation effect. Denote the $k$-th of the $p$ mediators (i.e. lipid species or metabolites) as ${M}_{k}$ and covariates as Z, the model is formulated as

$$MBD={c}^{*}+BMI\times {\gamma }^{*}+Z\times {\delta }_{y}^{*}+{\epsilon }_{1},$$

$${M}_{k}={c}_{k}+BMI\times {\alpha }_{k}+Z\times {\delta }_{k}+{e}_{k}, k=1,\dots , p,$$

$$MBD=c+BMI\times \gamma +{\sum }_{k=1}^{p}{M}_{k}{\times \beta }_{k}+Z\times {\delta }_{y}+{\epsilon }_{2},$$

where ${\alpha }_{k}{\prime}s$ are the coefficients of the association of the BMI exposure with mediators, ${\beta }_{k}$’s are the coefficients of the association of the mediators with MBD, ${\gamma }^{*}$ and $\gamma$ are the “total effect” and “direct effect” of the exposure on the outcome respectively, and ${\delta }_{y}^{*}, {\delta }_{k}, {\delta }_{y}$ are the coefficients of the association of the covariates and the outcome without adjusting for the mediators, the mediators, and the outcome adjusting for the mediators, respectively. The variables adjusted for include age, race, and family history of breast cancer for BMI at age 10, and additionally age at menarche and BMI at age 10 for BMI at age 18. HIMA adjusts for multiple comparisons by Bonferroni’s method to control the false discovery rate (FDR). We use the FDR *p*-value < 0.1 as a cut point to identify lipid species and metabolites that significantly mediate the associations between BMI exposures and MBD. Moreover, we quantify the magnitude of the mediation effects by calculating the proportion of total effects being mediated by the $k$-th mediator as ${\alpha }_{k}{\beta }_{k}/{\gamma }^{*}$.

## Results

Characteristics of the study population were previously published, but briefly, the average age of participants was 46 years old, and the majority of women identified as non-Hispanic White (~ 72%) [17]. After imputation, mean BMI was 17.4 kg/m^2^ and 22.1 kg/m^2^ at age 10 and age 18, respectively. Having experienced menarche below the median age (< 13 years old) was associated with a slightly higher BMI at ages 10 and 18. (Table 1) Non-Hispanic black women had higher BMI at age 18 than non-Hispanic White women. (Table 1).

**Table 1:** Characteristics of premenopausal women recruited during annual screening mammogram by body mass index at ages 10 and 18

		BMI (kg/m^2^) at age 10	BMI (kg/m^2^) at age 18
	N	Mean (SD)	Mean (SD)
Overall	700	17.4	22.1
Age, year			
< 46 years old	336	17.7 (3.0)	22.4 (4.7)
≥ 46 years old	364	17.1 (2.6)	21.8 (4.1)
Age at menarche, year			
< 13 years old	345	17.7 (2.9)	22.9 (4.7)
≥ 13 years old	355	17.0 (2.7)	21.3 (4.0)
Race			
Non-Hispanic white	507	17.3 (2.7)	21.7 (4.0)
Non-Hispanic black	162	17.8 (3.2)	23.5 (5.5)
Other Race	31	16.8 (2.0)	21.5 (3.4)
Family history of breast cancer			
Yes	153	17.7 (2.9)	22.2 (4.6)
No	547	17.3 (2.8)	22.0 (4.4)

*BMI* Body mass index, *N* Number, *SD* Standard deviation

### Associations of BMI at ages 10 and 18 across metabolites

**Fig. 1:**
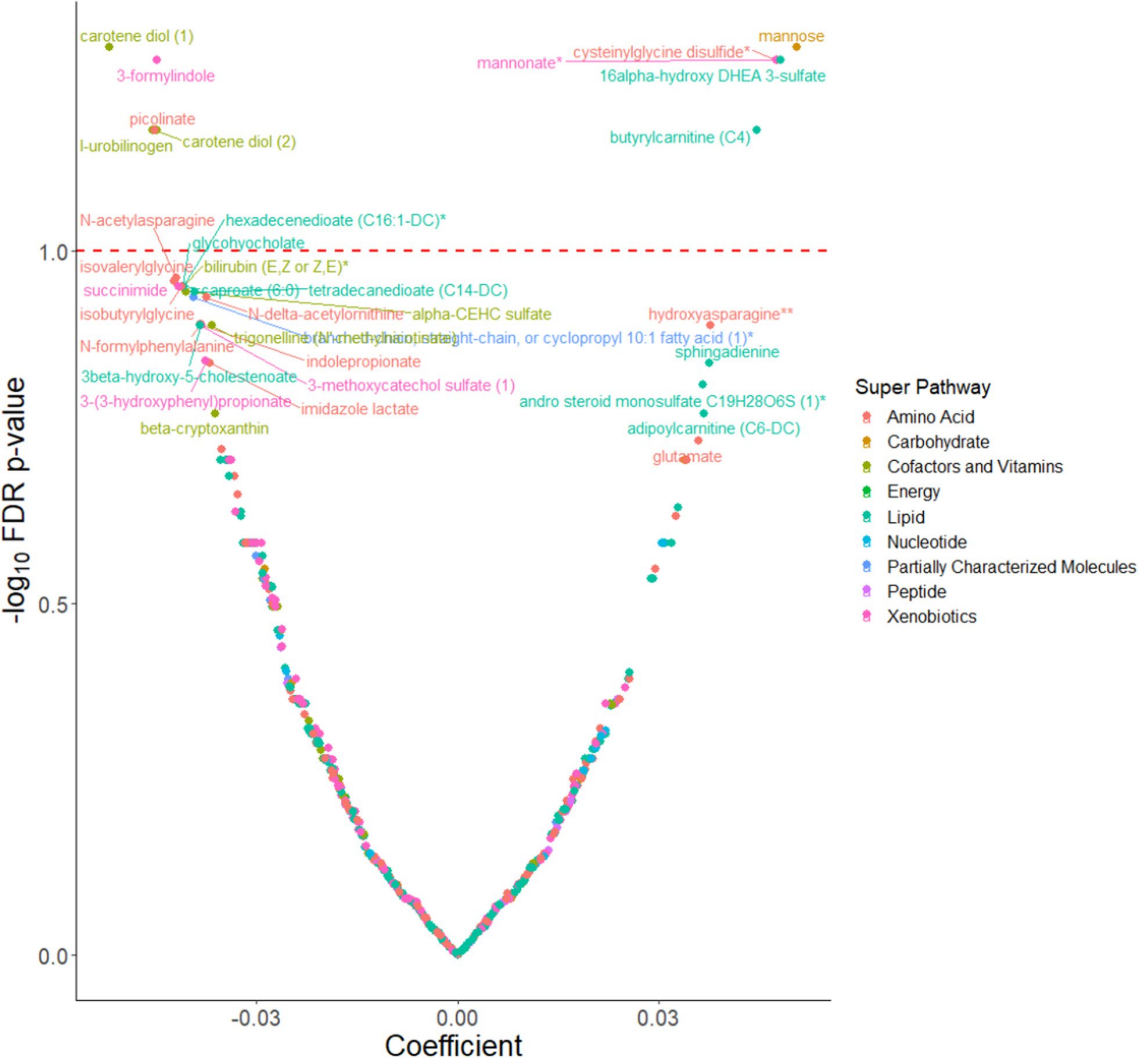
Covariate adjusted associations between metabolites and body mass index at age 10^a^. ^a^Model was adjusted for age at enrollment, race (non-Hispanic white, non-Hispanic black and other), and family history of breast cancer (yes, no). *FDR* False Discovery Rate

**Fig. 2:**
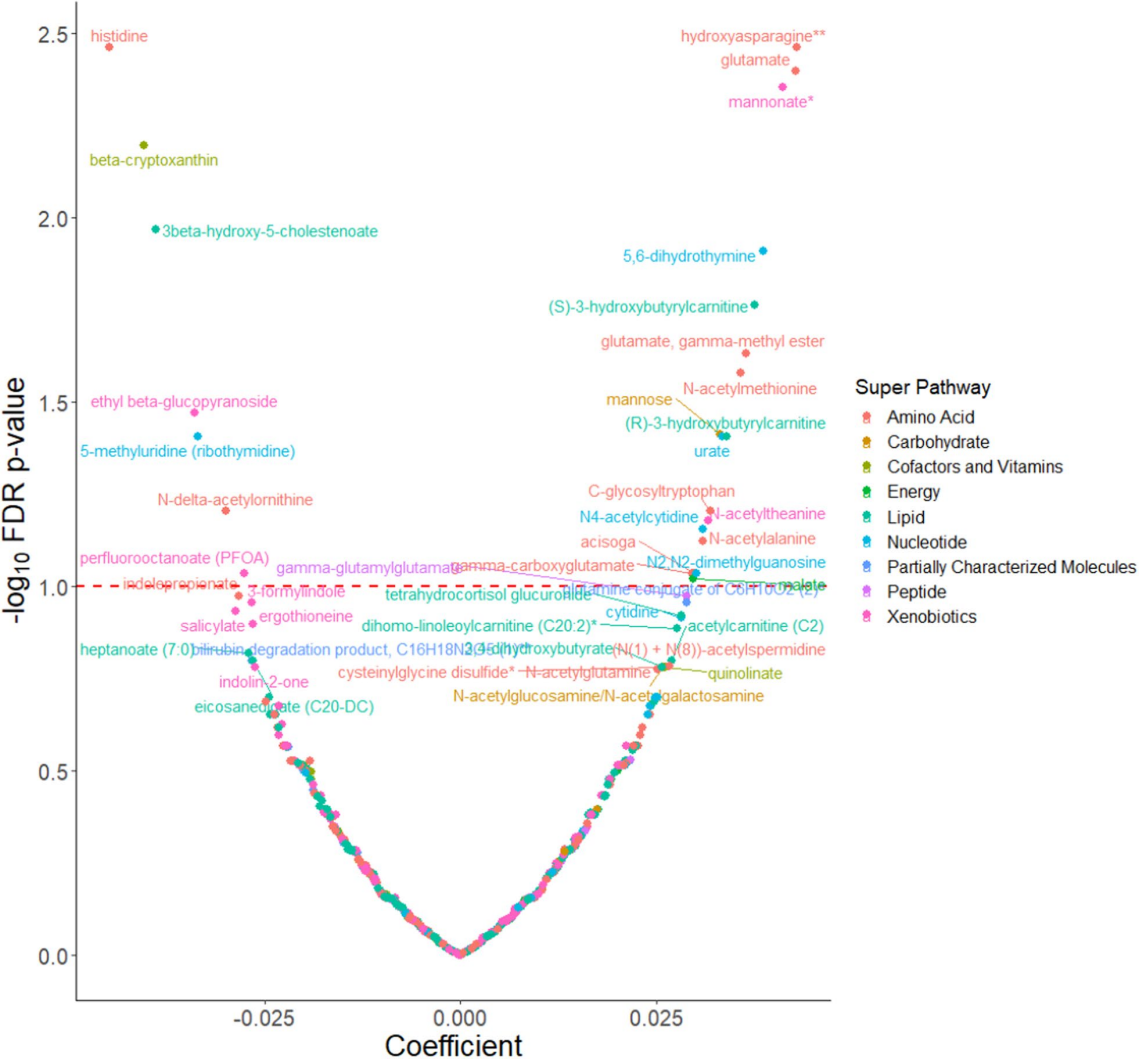
Covariate adjusted associations between metabolites and body mass index at age 18^a^. ^a^Model was adjusted for age at enrollment, race (non-Hispanic white, non-Hispanic black and other), family history of breast cancer (yes, no), BMI at age 10, and age at menarche. *FDR* False Discovery Rate

BMI at age 10 was significantly associated with 10 metabolites. Five metabolites (mannose, mannonate, cysteinylglycine disulfide, 16alpha-hydroxy DHEA 3-sulfate, and butyrylcarnitine (C4)) were positively associated and 5 metabolites (carotene diol (1), 3-formylindole, carotene diol (2), I-urobilinogen and picolinate) were inversely associated (Fig. 1). A total of 25 (18 positive and 7 inverse) metabolites were associated with BMI at age 18. The metabolites were from the amino acid (N = 10), nucleotide (N = 5), lipid (N = 3), xenobiotic (N = 4), vitamin/cofactor (N = 1), carbohydrate (N = 1), and energy (N = 1) super pathways (Fig. 2).

### Associations of BMI at ages 10 and 18 across lipid species

**Fig. 3:**
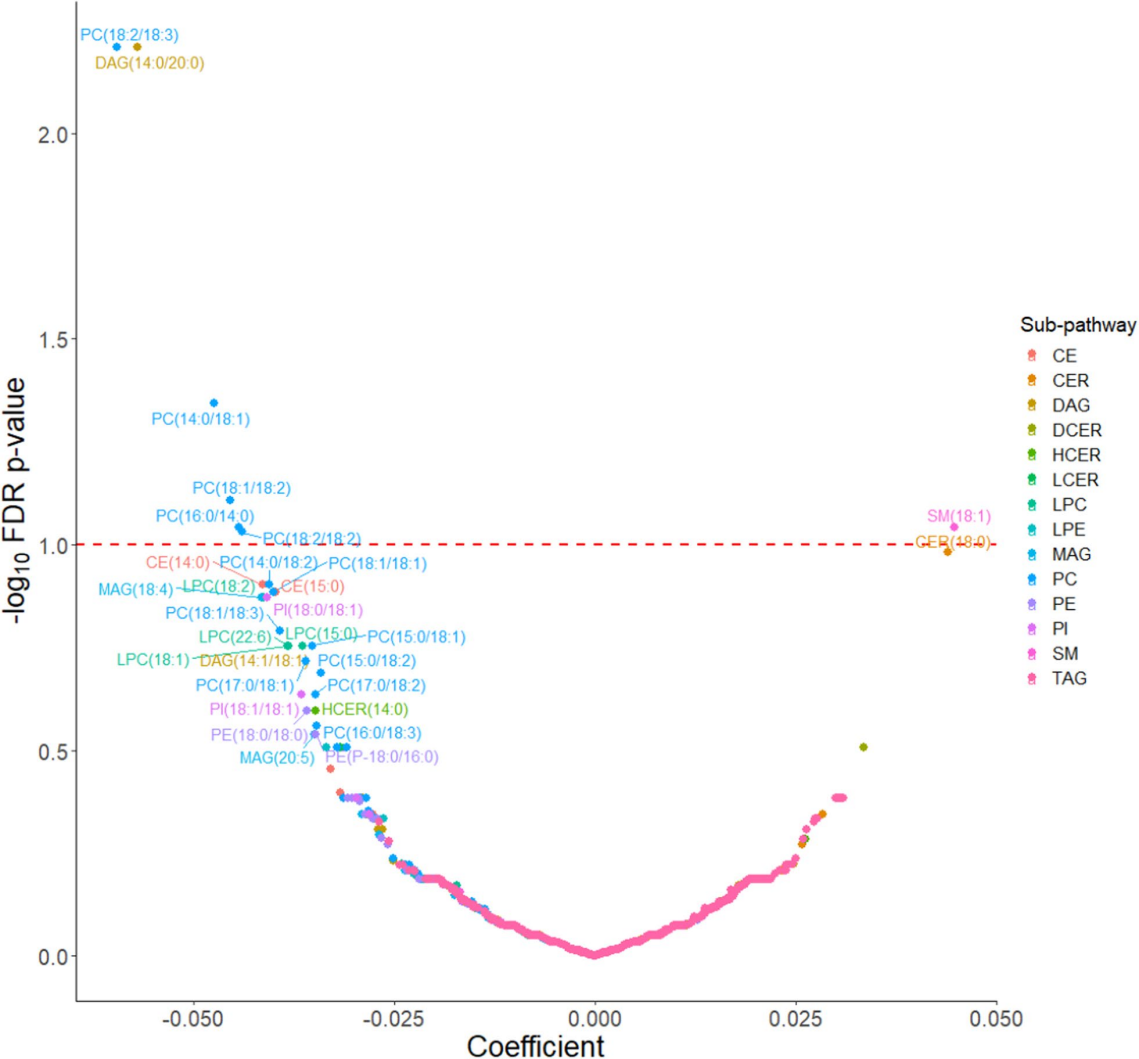
Covariate adjusted associations between lipid species and body mass index at age 10^a^. ^a^Model was adjusted for age at enrollment, race (non-Hispanic white, non-Hispanic black and other), and family history of breast cancer (yes, no). *PC* Phosphatidylcholine, *LPC* Lysophosphatidylcholine, *PE* Phosphatidylethanolamine, *LPE* Lysophosphatidylethanolamine, *PI* Phosphatidylinositol, *CER* Ceramide, *DCER* Dihydroceramide, *HCER* Hexosylceramide, *LCER* Lactosylceramide, *SM* Sphingomyelin, *CE* Cholesteryl ester, *DAG* Diacylglycerol, *TAG* Triacylglycerol, *MAG* Monoacylglycerol, *FDR* False discovery rate

**Fig. 4:**
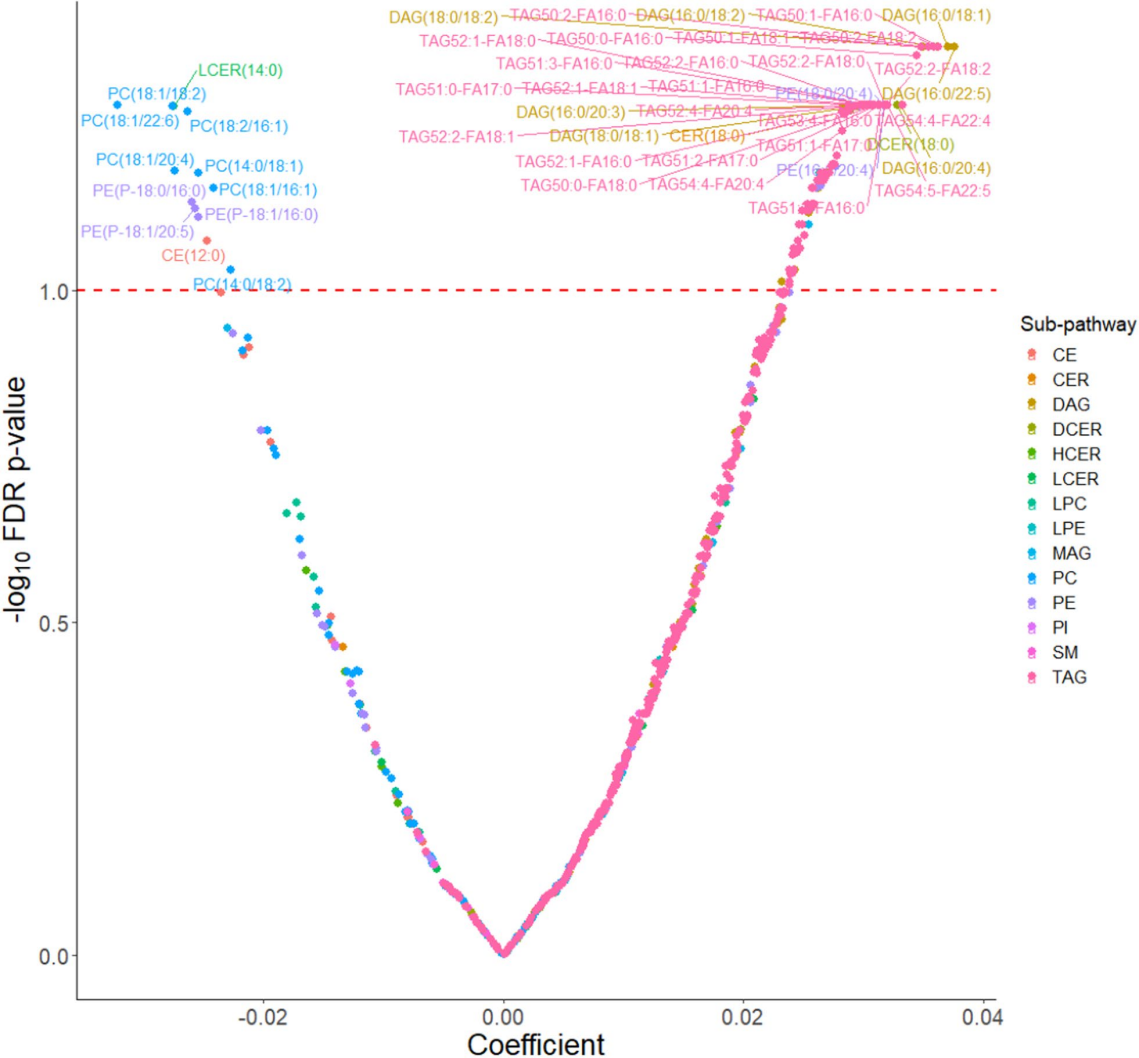
Covariate adjusted associations between lipid species and body mass index at age 18^a^. ^a^Model was adjusted for age at enrollment, race (non-Hispanic white, non-Hispanic black and other), family history of breast cancer (yes, no), BMI at age 10, and age at menarche. *PC* Phosphatidylcholine, *LPC* Lysophosphatidylcholine, *PE* Phosphatidylethanolamine, *LPE* Lysophosphatidylethanolamine, *PI* Phosphatidylinositol, *CER* Ceramide, *DCER* Dihydroceramide, *HCER* Hexosylceramide, *LCER* Lactosylceramide, *SM* Sphingomyelin, *CE* Cholesteryl ester, *DAG* Diacylglycerol, *TAG* Triacylglycerol, *MAG* Monoacylglycerol, *FDR* False discovery rate

BMI at age 10 was significantly associated with 7 lipid species; 6 inversely: (diacylglycerol (DAG(14:0/20:0)), (phosphatidylcholine (PC(18:2/18:3)), PC(14:0/18:1), PC(18:1/18:2), PC(16:0/14:0), PC(18:2/18:2)) and 1 positively: (sphingomyelin (SM(18:1))) (FDR *p*-value < 0.1) (Fig. 3). BMI at age 18 was associated with 139 lipid species (127 positive associations, mostly neutral complex lipids (N = 111); and 12 inverse associations, mostly phospholipids (N = 10)). (Fig. 4).

### Metabolites mediate the associations of BMI at ages 10 and 18 with MBD

Four metabolites from the amino acid (glutamate), vitamin/cofactors (β-cryptoxanthin), xenobiotic (phytanate), and lipid (cortolone glucuronide (1)) super pathways mediated the association of BMI at age 10 with VPD; and 2 metabolites (glutamate, β-cryptoxanthin) mediated the association of BMI at age 18 with VPD (Table 2). Glutamate was the strongest mediator across time points. Glutamate mediated 6.7% (FDR *p*-value = 0.06) and 9.3% (FDR *p*-value = 0.008) of the total effect of BMI at age 10 and 18, respectively, on VPD. β-cryptoxanthin mediated 4.1% (FDR *p*-value = 0.06) and 6.3% (FDR *p*-value = 0.04) of the total effect of BMI at age 10 and 18, respectively, on VPD.

**Table 2:** Metabolites mediating the association between body mass index at ages 10 and 18 with mammographic breast density

Metabolite	$\hat{\user2{\alpha }}$ ^c^	$\hat{\user2{\beta }}$ ^d^	% Total Effect^e^	FDR *p*-value	Super pathway
VPD					
BMI at age 10^a^					
Glutamate	0.036	− 0.049	6.65	0.06	Amino Acid
β-cryptoxanthin	− 0.036	0.030	4.09	0.06	Cofactors/Vitamins
Cortolone glucuronide (1)	0.033	− 0.016	2.03	0.07	Lipids (2)
Phytanate	− 0.034	0.020	2.58	0.06	Xenobiotics
BMI at age 18^b^					
Glutamate	0.043	− 0.039	9.29	0.008	Amino Acids
β-cryptoxanthin	− 0.040	0.028	6.34	0.04	Cofactors/Vitamins
NDV					
BMI at age 10^a^					
Citrulline	− 0.031	− 0.012	1.56	0.08	Amino Acids
Phytanate	− 0.034	− 0.050	7.10	0.05	Xenobiotics
Mannose	0.050	0.047	10.11	0.004	Carbohydrates
Glycerol	0.029	0.039	4.72	0.09	Lipids (1)
Isovalerylglycine	− 0.042	− 0.035	6.30	0.02	Amino Acids
Cysteinylglycine disulfide*	0.048	0.022	4.48	0.03	Amino Acids
Cortolone glucuronide (1)	0.033	0.051	7.10	0.07	Lipids (2)
Hydroxyasparagine**	0.038	0.047	7.51	0.04	Amino Acids
2,6-dihydroxybenzoic acid	− 0.029	− 0.011	1.35	0.08	Xenobiotics
Tetrahydrocortisone glucuronide (5)	0.032	− 0.049	− 6.65	0.08	Lipids (2)
BMI at age 18^b^					
Mannose	0.033	0.050	8.01	0.006	Carbohydrates
Urate	0.034	0.022	3.56	0.02	Nucleotide
Glycerol	0.024	0.024	2.76	0.08	Lipids (1)
N2,N5-diacetylornithine	− 0.023	− 0.035	3.83	0.09	Amino Acids
2,6-dihydroxybenzoic acid	− 0.023	− 0.003	0.30	0.08	Xenobiotics

^a^Model was adjusted for age at enrollment, race (non-Hispanic white, non-Hispanic black and other), family history of breast cancer (yes, no)

^b^Model was adjusted for age at enrollment, race (non-Hispanic white, non-Hispanic black and other), family history of breast cancer (yes, no), BMI at age 10, and age at menarche

^c^$\hat{\alpha }$ is the coefficient of the relationship between the exposure (BMI measure) and mediator (metabolite)

^d^$\hat{\beta }$ is the coefficient of the relationship between the mediator (metabolite) and outcome (mammographic breast density) adjusted for the exposure (BMI measure)

^e^% Total Effect= $\widehat{\alpha }$ * $\widehat{\beta }$**/**$\upgamma$, where $\upgamma$ is the coefficient of the relationship between the exposure (BMI measure) and the outcome (mammographic breast density). *BMI* Body mass index, *FDR* False discovery rate, *NDV* Non-dense volume, *VPD* Volumetric percent density

Ten metabolites and 5 metabolites significantly mediated the associations of BMI at ages 10 and 18 with NDV, respectively. Four of the 10 metabolites that mediated the association of BMI at age 10 with NDV are from the amino acid super pathway (citrulline, isovalerylglycine, cysteinylglycine disulfide, and hydroxyasparagine), 3 are from the lipid super pathway (glycerol, cortolone glucuronide (1) and tetrahydrocortisone glucuronide (5)), 2 are from the xenobiotic super pathway (phytanate and 2,6-dihydroxybenzoic acid) and 1 from the carbohydrate super pathway (mannose). Mannose, glycerol, and 2,6-dihydroxybenzoic acid also significantly mediated the association of BMI at age 18 with NDV, as well as urate (nucleotide super pathway) and N2,N5-diacetylornithine (amino acid super pathway). Mannose was the strongest mediator for both time points; it mediated 10.1% of the total effect of BMI at age 10 and NDV, FDR *p*-value = 0.004 and 8.0% of the total effect of BMI at age 18 with NDV, FDR *p*-value = 0.006 (Table 2). No significant associations were observed for DV.

### Lipid species mediate the associations of BMI at ages 10 and 18 with MBD

The association of BMI at age 10 with VPD was significantly mediated by 4 lipid species; 2 of the species were from the phospholipid super pathway (LPC(18:1) and PC(18:1/18:1)), 2 were from the sphingolipid super pathway (ceramide (CER(18:0)) and (lactosylceramide (LCER(14:0)). The strongest mediator was PC(18:1/18:1), which mediated 9.7% of the total effect of BMI at age 10 and VPD, FDR *p*-value = 0.009. Additionally, CER(18:0), LCER(14:0), and LPC(18:1) mediated 8.7% (FDR *p*-value = 0.009), 7.9% (FDR *p*-value = 0.03) and 1.6% (FDR *p*-value = 0.08) of the association of BMI at age 10 with VPD, respectively. Five lipid species mediated the association of BMI at age 18 with VPD, including CER(18:0), LCER(14:0), PC(18:1/18:1), TAG56:5-FA22:5, and TAG52:2-FA16:0. TAG56:5-FA22:5 was the strongest—mediating 16.1%, (FDR *p*-value = 0.01) of the association between BMI at age 18 and VPD (Table 3).

**Table 3:** Lipid species mediating the association between body mass index at ages 10 and 18 with mammographic breast density

	$\hat{\user2{\alpha }}$ ^c^	$\hat{\user2{\beta }}$ ^d^	% Total Effect^e^	FDR *p*-value
VPD				
BMI at age 10^a^				
CER(18:0)	0.044	− 0.052	8.73	0.009
LCER(14:0)	− 0.032	0.065	7.86	0.03
LPC(18:1)	− 0.038	0.011	1.59	0.08
PC(18:1/18:1)	− 0.040	0.064	9.72	0.009
BMI at age 18^b^				
CER(18:0)	0.030	− 0.059	10.07	0.01
LCER(14:0)	− 0.027	0.053	8.14	0.01
PC(18:1/18:1)	− 0.021	0.064	7.66	0.04
TAG56:5-FA22:5	0.027	0.106	− 16.14	0.01
TAG52:2-FA16:0	0.031	− 0.062	10.79	0.008
NDV				
BMI at age 10^a^				
CER(18:0)	0.044	0.044	8.21	0.01
LPC(18:1)	− 0.038	− 0.038	6.09	0.01
PC(18:1/18:1)	− 0.040	− 0.071	11.92	0.01
BMI at age 18^b^				
CER(18:0)	0.030	0.039	5.65	0.01
DAG(16:0/18:1)	0.037	0.062	11.02	0.002
PC(18:1/18:1)	− 0.021	− 0.080	8.13	0.04
TAG49:2-FA18:2	0.023	− 0.073	− 8.06	0.04
TAG56:6-FA22:5	0.022	− 0.128	− 13.82	0.04
TAG56:6-FA20:4	0.023	0.059	6.57	0.04

^a^Model was adjusted for age at enrollment, race (non-Hispanic white, non-Hispanic black and other), family history of breast cancer (yes, no)

^b^Model was adjusted for age at enrollment, race (non-Hispanic white, non-Hispanic black and other), family history of breast cancer (yes, no), BMI at age 10, and age at menarche

^c^$\hat{\alpha }$ is the coefficient of the relationship between the exposure (BMI measure) and mediator (lipid species)

^d^$\hat{\beta }$ is the coefficient of the relationship between the mediator (lipid species) and outcome (mammographic breast density) adjusted for the exposure (BMI measure). *BMI* Body mass index, *FDR* False Discovery rate, *CER* Ceramide, *DAG* Diacylglycerol, *LCER* Lactosylceramide, *LPC* Lysophosphatidylcholine, *NDV* Non-dense volume, *PC* Phosphatidylcholine, *TAG* Triacylglycerol, *VPD* Volumetric percent density

^e^% Total Effect= $\widehat{\alpha }$ * $\widehat{\beta }$**/**$\upgamma$, where $\upgamma$ is the coefficient of the relationship between the exposure (BMI measure) and the outcome (mammographic breast density)

PC(18:1/18:1), the strongest mediator of the association of BMI at age 10 with NDV, mediating 11.9% of the total effect (FDR *p*-value = 0.01) (Table 3). Six lipid species (CER(18:0), DAG(16:0/18:1), PC(18:1/18:1), TAG49:2-FA18:2, TAG56:6-FA22:5, TAG56:6-FA20:4) mediated the association ofBMI at age 18 and NDV with TAG56:6-FA22:5 (− 13.8%, FDR *p*-value = 0.04) and DAG(16:0/18:1; 11.0%, FDR *p*-value = 0.002), being the strongest mediators. (Table 3). No lipid species mediated the associations of BMI at ages 10 and 18 with DV, which is validated by our previous study that demonstrated no significant associations of the lipidome and DV [17].

## Discussion

We identified metabolites, specifically glutamate and β-cryptoxanthin and lipid species, PC(18:1/18:1), CER(18:0), and LCER (14:0) that mediated the associations of BMI at ages 10 and 18 with VPD. This is the first study, to our knowledge, to perform a comprehensive lipodomic analysis to investigate the mediating role of lipid species on the associations of BMI in childhood with MBD. As well the first to use a high dimensional approach to investigate the mediating role of metabolites on the associations of BMI at 10 and 18 with MBD.

A previous study in 182 young women (25–29 years) reported that that an unnamed metabolite, “X-16576”, mediated the relationship between childhood adiposity and percent dense volume [18]. Our study brings important new insights by utilizing a high dimensional approach among a larger study population (N = 700 vs N = 182) of premenopausal women attending annual screening mammogram [18]. Further, our high-dimensional analysis and more complete characterization of the metabolites could have enabled us to identify metabolites that they did not observe in their analysis. For instance, 230 of the 880 biochemicals they profiled in their study were unnamed biochemicals. Also, metabolomics is an evolving field in which new metabolites are identified regularly; the metabolites included in the untargeted global panel at the time of our study may differ from other studies.

The metabolite that most strongly mediated the relationships between BMI at ages 10 and 18 in our study was glutamate. Glutamate was positively associated with BMI at age 10 and age 18, but inversely associated with VPD. These findings are consistent with several studies where glutamate was positively associated with various measures of adiposity in adults.[6–8, 28], as well as among adolescents/children [29, 30]. Glutamate is positively associated with visceral adipose tissue and metabolic syndrome [31]. Studies by Maltais-Payette et al. suggest branched-chain-amino-acid (BCAA) catabolism may play a role in this relationship because glutamate is a by-product of this process [31, 32]. Glutamate is produced by various tissues in the body, including adipose tissue. An animal study found that obese mice produced higher levels of glutamate from their adipose tissue compared to lean mice, but similar amounts of glutamate were produced from other tissues [33]. It is possible that the relationship between BMI and glutamate may reflect similarly to the composition of breast tissue, given that NDV in the breast mainly consists of fatty tissue.

β-cryptoxanthin also significantly mediated the relationship between BMI at ages 10 and 18 with VPD but was inversely associated with BMI at ages 10 and 18, and positively associated with VPD. A similar inverse relationship between β-cryptoxanthin and BMI was also identified in the McClain et al. [6] study. A study of postmenopausal women found that provitamin A carotenoids, including β-cryptoxanthin, were strongly, inversely associated with BMI even when controlling for total energy intake [34]. β-cryptoxanthin can be found in citrus, and results from an animal study where β-cryptoxanthin was administered to mice found a reduction in adipocytes as well as an immune and inflammatory response [35]. This relationship is further validated by a trial performed in Japanese women who consumed β-cryptoxanthin supplement and, although did not see a difference in weight, found changes in adipocytokines [36]. Indications of the potential anti-inflammatory properties of β-cryptoxanthin support the inverse relationship with BMI, but further research is necessary to explore how β-cryptoxanthin mediates the relationship between BMI and MBD.

LPC(18:1) mediated 1.59% of the total effect of BMI at age 10 and VPD, FDR *p*-value = 0.09. Although this is modest mediation compared to the other lipid species, LPC(18:1) has been consistently identified as being inversely associated with BMI and adiposity among adults and adolescents/children [10–13, 37]. Studies suggest the inverse association of LPC with obesity may be related to the transfer of PC to cholesterol by lecithin-cholesterol acyltransferase (LCAT) or LPC catabolism, resulting in lower levels of LPC present in the blood [11, 37]. Findings from both mediation analyses imply potential inflammatory mechanisms as well as the possible influence of metabolic dysregulation. Additional research to further elucidate the underlying biological mechanism behind these associations is necessary.

### Strengths and limitations

Our study has many strengths including a relatively large and diverse study population. We are also the first to our knowledge to investigate the mediating role of lipid species and metabolites on the association of childhood/early adulthood adiposity with MBD utilizing a high dimensional mediation analysis approach. This method is unique because it considers the relationship between the mediators when calculating the total percent mediated. We collected data on reproductive, demographic, and behavioral characteristics that were used as confounders in our analysis.

Although this study has many strengths, it has some limitations. BMI at ages 10 and 18 are both based on self-reported measures, the Stunkard figure rating scale, and self-reported weight from age 18. Although self-reported measures of adiposity are often underestimated, they are often highly correlated with measured weight and BMI, this is consistent with adults recalling BMI from childhood as well [38, 39]. The Stunkard pictogram has been validated in many studies and found to be a reliable estimate of BMI at age 10 [40–42]. For instance, a validation study from a Boston-area longitudinal study of school children reported a high correlation between participants' adult-recalled body size at age 10 and their measured BMI at age 10 (*r* = 0.65). We have also validated it in our previous studies within the same study population and observed strong positive correlations between adiposity at age 10 and BMI at age 18. Further, a systematic review and meta-analysis that explored the validity of early-life recall of BMI reported strong correlations with prospective measures with a mean pooled difference of only 0.06 kg/m^2^ (95% CI − 0.62–0.73) between recalled BMI and prospective measures [39]. Another potential limitation is that BMI may not reflect an accurate measure of adiposity due to differences in body composition, but findings from a study that compared the metabolomic profiling of BMI to percent body fat and fat mass of the body found strong correlations across many metabolites and suggests that BMI may be a good proxy for measures of adiposity such as body fat percent [43]. Also, BMI and body fat percent that were measured at study initiation were highly correlated in our study participants (r = 0.88).

Metabolites and lipid species were measured at a single time point, which may not provide an accurate depiction of longitudinal exposure over time. Also, the blood sample provided for metabolomic and lipidomic analysis was collected on the same day as mammographic imaging which may impact temporal associations. Nevertheless, we evaluated the associations of early-life adiposity measures which were collected via recall, rather than current BMI. Although participants provided this information on the day of their mammogram, they do not reflect the BMI of the women on the day the mammogram was performed. We acknowledge that this approach still has limitations, and our findings will need to be interpreted within the context of using recalled data, which has been validated. The ideal study would be one where samples are collected from girls at ages 10 and 18, stored for several years and they are then followed for 30–40 years when they undergo screening mammogram. This approach is however challenging in the real setting; hence, our study helps to bridge fundamental gaps.

Lastly, we did not assess for the potential interaction between the exposure and mediators since we utilized high dimensional data for the mediators (metabolites N = 828 and lipid species N = 857) and there is also the possibility of residual or unmeasured confounding that was not controlled for in the analyses because models with the same exposure used the same covariate set.

## Conclusions

Metabolites in amino acid, lipid, cofactor/vitamin, and xenobiotic super-pathways as well as lipid species in phospholipid, neutral complex lipid and sphingolipid super-pathways mediate the associations of early-life BMI with VPD/NDV in premenopausal women. This innovative study offers insight into the biological mechanisms underlying the associations of early-life adiposity and MBD, and can support future research into breast cancer prevention.

## Data Availability

Data used/analyzed are not publicly available but can be requested from the corresponding author.

## Abbreviations

MBD: Mammographic breast density
PC: Phosphatidylcholines
LPC: Lysophosphatidylcholines
PE: Phosphatidylethanolamines
LPE: Lysophosphatidylethanolamines
PI: Phosphatidylinositols
CER: Ceramides
DCER: Dihydroceramides
HCER: Hexosylceramides
LCER: Lactosylceramides
SM: Sphingomyelins
CE: Cholesteryl esters
DAG: Diacylglycerols
TAG: Triacylglycerols
MAG: Monoacylglycerols
VPD: Volumetric percent density
DV: Dense volume
NDV: Non-dense volume
BMI: Body mass index
FDR: False discovery rate
SD: Standard deviation
CI: Confidence interval

## References

[1] Bissell MCS, Kerlikowske K, Sprague BL, Tice JA, Gard CC, Tossas KY, et al. Breast cancer population attributable risk proportions associated with body mass index and breast density by race/ethnicity and menopausal status. Cancer Epidemiol Biomarkers Prev. 2020;29:2048–56. 32727722 10.1158/1055-9965.EPI-20-0358 PMC7541499

[2] Alimujiang A, Imm KR, Appleton CM, Colditz GA, Berkey CS, Toriola AT. Adiposity at age 10 and mammographic density among premenopausal women. Cancer Prev Res. 2018;11:287–94. 10.1158/1940-6207.CAPR-17-0309 PMC7473460 29500187

[3] Alimujiang A, Appleton C, Colditz GA, Toriola AT. Adiposity during early adulthood, changes in adiposity during adulthood, attained adiposity, and mammographic density among premenopausal women. Breast Cancer Res Treat. 2017;166:197–206. 10.1007/s10549-017-4384-4. 28702890 10.1007/s10549-017-4384-4

[4] Moore JX, Han Y, Appleton C, Colditz G, Toriola AT. Determinants of mammographic breast density by race among a large screening population. JNCI Cancer Spectr. 2020. 10.1093/jncics/pkaa010/5758266. 32373777 10.1093/jncics/pkaa010 PMC7192029

[5] Dettmer K, Aronov PA, Hammock BD. Mass spectrometry-based metabolomics. Mass Spectrom Rev. 2007;26:51–78. 16921475 10.1002/mas.20108 PMC1904337

[6] Mcclain KM, Friedenreich CM, Matthews CE, Sampson JN, Check DP, Brenner DR, et al. Body composition and metabolomics in the Alberta physical activity and breast cancer prevention trial. J Nutr. 2022;152:419–28. 10.1093/jn/nxab388. 34791348 10.1093/jn/nxab388 PMC8826845

[7] Stevens VL, Carter BD, McCullough ML, Campbell PT, Wang Y. Metabolomic profiles associated with BMI, waist circumference, and diabetes and inflammation biomarkers in women. Obesity. 2020;28:187–96. 31777189 10.1002/oby.22670

[8] Carayol M, Leitzmann MF, Ferrari P, Zamora-Ros R, Achaintre D, Stepien M, et al. Blood metabolic signatures of body mass index: a targeted metabolomics study in the EPIC cohort. J Proteome Res. 2017;16:3137–46. 28758405 10.1021/acs.jproteome.6b01062 PMC6198936

[9] Quehenberger O, Dennis EA. The human plasma lipidome. N Engl J Med. 2011;365:1812–23. 22070478 10.1056/NEJMra1104901 PMC3412394

[10] Gerl MJ, Klose C, Surma MA, Fernandez C, Melander O, Männistö S, et al. Machine learning of human plasma lipidomes for obesity estimation in a large population cohort. PLoS Biol. 2019;17:1–25. 10.1371/journal.pbio.3000443 PMC6799887 31626640

[11] Rauschert S, Uhl O, Koletzko B, Kirchberg F, Mori TA, Huang RC, et al. Lipidomics reveals associations of phospholipids with obesity and insulin resistance in young adults. J Clin Endocrinol Metab. 2016;101:871–9. 26709969 10.1210/jc.2015-3525

[12] Yin R, Wang X, Li K, Yu K, Yang L. Lipidomic profiling reveals distinct differences in plasma lipid composition in overweight or obese adolescent students. BMC Endocr Disord. 2021;21:1–10. 34641844 10.1186/s12902-021-00859-7 PMC8513241

[13] Wang Y, Jiang CT, Song JY, Song QY, Ma J, Wang HJ. Lipidomic profile revealed the association of plasma lysophosphatidylcholines with adolescent obesity. Biomed Res Int. 2019;2019:1–9. 10.1155/2019/1382418 PMC6930386 31915678

[14] Beyene HB, Olshansky G, Smith AAT, Giles C, Huynh K, Cinel M, et al. High-coverage plasma lipidomics reveals novel sex-specific lipidomic fingerprints of age and BMI: evidence from two large population cohort studies. PLoS Biol. 2020. 10.1371/journal.pbio.3000870. 32986697 10.1371/journal.pbio.3000870 PMC7544135

[15] Matthew KA, Getz KR, Jeon MS, Luo C, Luo J, Toriola AT. Associations of vitamins and related cofactor metabolites with mammographic breast density in premenopausal women. J Nutr. 2024;154:424–34. 38122846 10.1016/j.tjnut.2023.12.023 PMC10900193

[16] Jung S, Silva S, Dallal CM, LeBlanc E, Paris K, Shepherd J, et al. Untargeted serum metabolomic profiles and breast density in young women. Cancer Causes Control. 2024;35:323–34. 10.1007/s10552-023-01793-w. 37737303 10.1007/s10552-023-01793-w

[17] Getz KR, Jeon MS, Luo C, Luo J, Toriola AT. Lipidome of mammographic breast density in premenopausal women. Breast Cancer Res. 2023;25:1–13. 10.1186/s13058-023-01725-1. 37814330 10.1186/s13058-023-01725-1 PMC10561435

[18] Dorgan JF, Baer HJ, Bertrand KA, LeBlanc ES, Jung S, Magder LS, et al. Childhood adiposity, serum metabolites and breast density in young women. Breast Cancer Res. 2022;24:1–11. 10.1186/s13058-022-01588-y. 36536390 10.1186/s13058-022-01588-y PMC9764542

[19] Toriola AT, Appleton CM, Zong X, Luo J, Weilbaecher K, Tamimi RM, et al. Circulating receptor activator of nuclear factor-κB (RANK), RANK ligand (RANKL), and mammographic density in premenopausal women. Cancer Prev Res. 2018;11:789–96. 10.1158/1940-6207.CAPR-18-0199 PMC6784533 30352839

[20] Berkey CS, Rockett HRH, Field AE, Gillman MW, Frazier AL, Camargo CA, et al. Activity, dietary intake, and weight changes in a longitudinal study of preadolescent and adolescent boys and girls. Pediatrics. 2000;105:e56–e56. 10742377 10.1542/peds.105.4.e56

[21] Metabolon. Complex Lipids Targeted Panel. [cited 2023 May 18]. Available from: https://www.metabolon.com/lp/complex-lipid-panel/

[22] Hastie T, Tibshirani R, Narasimhan B, Chu G. Impute: impute: imputation for microarray data_.R package version 1.70.0. 2022.

[23] Collet TH, Sonoyama T, Henning E, Keogh JM, Ingram B, Kelway S, et al. A metabolomic signature of acute caloric restriction. J Clin Endocrinol Metab. 2017;102:4486–95. 29029202 10.1210/jc.2017-01020 PMC5718701

[24] Troyanskaya O, Cantor M, Sherlock G, Brown P, Hastie T, Tibshirani R, et al. Missing value estimation methods for DNA microarrays. Bioinformatics. 2001;17:520–5. 11395428 10.1093/bioinformatics/17.6.520

[25] Johnson WE, Li C, Rabinovic A. Adjusting batch effects in microarray expression data using empirical Bayes methods. Biostatistics. 2007;8:118–27. 16632515 10.1093/biostatistics/kxj037

[26] van Buuren S, Groothuis-Oudshoorn K. Mice: multivariate imputation by chained equations in R. J Stat Softw. 2011. 10.18637/jss.v045.i03.

[27] Zhang H, Zheng Y, Zhang Z, Gao T, Joyce B, Yoon G, et al. Estimating and testing high-dimensional mediation effects in epigenetic studies. Bioinformatics. 2016;32:3150–4. 27357171 10.1093/bioinformatics/btw351 PMC5048064

[28] Moore SC, Matthews CE, Sampson JN, Stolzenberg-Solomon RZ, Zheng W, Cai Q, et al. Human metabolic correlates of body mass index. Metabolomics. 2014;10:259–69. 25254000 10.1007/s11306-013-0574-1 PMC4169991

[29] Butte NF, Liu Y, Zakeri IF, Mohney RP, Mehta N, Voruganti VS, et al. Global metabolomic profiling targeting childhood obesity in the Hispanic population. Am J Clin Nutr. 2015;102:256–67. 26085512 10.3945/ajcn.115.111872 PMC4515872

[30] Müllner E, Röhnisch HE, von Brömssen C, Moazzami AA. Metabolomics analysis reveals altered metabolites in lean compared with obese adolescents and additional metabolic shifts associated with hyperinsulinaemia and insulin resistance in obese adolescents: a cross-sectional study. Metabolomics. 2021;17:1–13. 33438144 10.1007/s11306-020-01759-y PMC7803706

[31] Maltais-Payette I, Boulet M-M, Prehn C, Adamski J, Tchernof A. Circulating glutamate concentration as a biomarker of visceral obesity and associated metabolic alterations. Nutr Metab. 2018;15:78. 10.1186/s12986-018-0316-5 PMC6219091 30450120

[32] Maltais-Payette I, Allam-Ndoul B, Pérusse L, Vohl M-C, Tchernof A. Circulating glutamate level as a potential biomarker for abdominal obesity and metabolic risk. Nutr Metab Cardiovasc Dis. 2019;29:1353–60. 31668457 10.1016/j.numecd.2019.08.015

[33] Nagao H, Nishizawa H, Bamba T, Nakayama Y, Isozumi N, Nagamori S, et al. Increased dynamics of tricarboxylic acid cycle and glutamate synthesis in obese adipose tissue: in vivo metabolic turnover analysis. J Biol Chem. 2017;292:4469–83. 28119455 10.1074/jbc.M116.770172 PMC5377766

[34] Chai W, Conroy SM, Maskarinec G, Franke AA, Pagano IS, Cooney RV. Associations between obesity and serum lipid-soluble micronutrients among premenopausal women. Nutr Res. 2010;30:227–32. 20534324 10.1016/j.nutres.2010.04.006 PMC2884001

[35] Takayanagi K, Morimoto S, Shirakura Y, Mukai K, Sugiyama T, Tokuji Y, et al. Mechanism of visceral fat reduction in Tsumura Suzuki obese, diabetes (TSOD) mice orally administered β-cryptoxanthin from Satsuma mandarin oranges (Citrus unshiu Marc). J Agric Food Chem. 2011;59:12342–51. 22085304 10.1021/jf202821u

[36] Iwamoto M, Imai K, Ohta H, Shirouchi B, Sato M. Supplementation of highly concentrated β-cryptoxanthin in a satsuma mandarin beverage improves adipocytokine profiles in obese Japanese women. Lipids Health Dis. 2012;11:52. 22584034 10.1186/1476-511X-11-52 PMC3475063

[37] Yin X, Willinger CM, Keefe J, Liu J, Fernández-Ortiz A, Ibáñez B, et al. Lipidomic profiling identifies signatures of metabolic risk. EBioMedicine. 2020;51:102520. 31877415 10.1016/j.ebiom.2019.10.046 PMC6938899

[38] McAdams MA, Van Dam RM, Hu FB. Comparison of self-reported and measured BMI as correlates of disease markers in US adults. Obesity. 2007;15:188–96. 17228047 10.1038/oby.2007.504

[39] De Rubeis V, Bayat S, Griffith LE, Smith BT, Anderson LN. Validity of self-reported recall of anthropometric measures in early life: a systematic review and meta-analysis. Obes Rev. 2019;20:1426–40. 31184422 10.1111/obr.12881

[40] Must A, Willett WC, Dietz WH. Remote recall of childhood height, weight, and body build by elderly subjects. Am J Epidemiol. 1993;138:56–64. 8333427 10.1093/oxfordjournals.aje.a116777

[41] Yochum L, Tamimi RM, Hankinson SE. Birthweight, early life body size and adult mammographic density: a review of epidemiologic studies. Cancer Causes Control. 2014;25:1247–59. 25053404 10.1007/s10552-014-0432-0

[42] Sellers TA, Vachon CM, Pankratz VS, Janney CA, Fredericksen Z, Brandt KR, et al. Association of childhood and adolescent anthropometric factors, physical activity, and diet with adult mammographic breast density. Am J Epidemiol. 2007;166:456–64. 17548785 10.1093/aje/kwm112

[43] Dorgan JF, Ryan AS, LeBlanc ES, Van Horn L, Magder LS, Snetselaar LG, et al. A comparison of associations of body mass index and dual-energy x-ray absorptiometry measured percentage fat and total fat with global serum metabolites in young women. Obesity. 2023;31:525–36. 36642094 10.1002/oby.23619 PMC9937438

